# The Impact of Obesity on the Association between Vitamin D Deficiency and Cardiovascular Disease

**DOI:** 10.3390/nu11102458

**Published:** 2019-10-14

**Authors:** Stavroula A. Paschou, Marinos Kosmopoulos, Ilias P. Nikas, Michael Spartalis, Evanthia Kassi, Dimitrios G. Goulis, Irene Lambrinoudaki, Gerasimos Siasos

**Affiliations:** 1 Division of Endocrinology and Diabetes, Aghia Sophia Hospital, Medical School, National and Kapodistrian University of Athens, 11527 Athens, Greece; 2 School of Medicine, European University Cyprus, 2404 Nicosia, Cyprus; i.nikas@euc.ac.cy; 3 Department of Medicine, Division of Cardiology, School of Medicine, University of Minnesota, Minneapolis, MN 55455, USA; marinos.kosmopoulos@outlook.com.gr; 4 Division of Cardiology, Onassis Cardiac Surgery Centre, 17674 Athens, Greece; msparta@med.uoa.gr; 5 Department of Biological Chemistry, Medical School, National and Kapodistrian University of Athens, 11527 Athens, Greece; evakassis@gmail.com; 6 Department of Obstetrics and Gynecology, Unit of Reproductive Endocrinology, Medical School, Aristotle University of Thessaloniki, 56403 Thessaloniki, Greece; dimitrios.goulis@gmail.com; 7 Department of Obstetrics and Gynecology, Medical School, National and Kapodistrian University of Athens, 11526 Athens, Greece; ilambrinoudaki@med.uoa.gr; 8 Department of Cardiology, Hippokration Hospital, Medical School, National and Kapodistrian University of Athens, 11526 Athens, Greece; gsiasos@med.uoa.gr

**Keywords:** vitamin D, obesity, cardiovascular disease, atherosclerosis

## Abstract

The aim of this article is to review the literature regarding the relationship between vitamin D deficiency and cardiovascular disease (CVD) and its modification in the presence of obesity. Despite the strong association between vitamin D status and cardiovascular outcomes, vitamin D supplementation trials in the general population have failed to decrease the incidence of cardiovascular events and mortality. A comprehensive study of the published literature and a comparison with experimental data lead to the conclusion that obesity, due to its high prevalence and strong association with both vitamin D deficiency and CVD, may act as a critical confounder, which is responsible for the different results on this association. Adoption of a vitamin D preventive supplementation strategy for CVD is unlikely to yield any benefit to the general population. However, it might be particularly useful in obese adults with increased risk for CVD.

## 1. Introduction

Vitamin D is a lipid-soluble secosteroid hormone that was initially described as a crucial mediator of calcium homeostasis [1]. In humans, it is mainly synthesized in the skin, and its formation is catalyzed by ultraviolet B irradiation. After its formation, vitamin D needs to be transformed into its active form, 1,25 dihydroxy vitamin D [1,25(OH)2D], with 25-hydroxy-vitamin D [25(OH)D] being the most abundant form and thus the most frequently indicator used to assess vitamin D concentrations. While, initially, the interest in vitamin D was focused on mineral distribution and bone health, it was soon discovered that its receptors are expressed in many different tissues, which thus raises the probability of vitamin D implication in other conditions. Vitamin D concentrations correlate well with many medical conditions, most notably, cardiovascular disease (CVD) [2,3], and with the incidence of colorectal carcinoma [4] and multiple sclerosis [5,6]. Another important finding in this direction is the discovery that a substantial proportion of the population is deficient in vitamin D, which has been defined as 25(OH)D concentration <20 ng/mL (<50 nmol/L) [7], while the concentration of 30 ng/mL has been recommended as a threshold [8]. The average recommended uptake is 5–20 μg/day [9]. As per both definitions and irrespectively of the country studied, deficiency affects a great proportion of the population, with an estimated prevalence of 34–66% [10], while there are reports of deficiency even in 94% of some population groups [11,12].

At the same time, the prevalence of obesity is rapidly increasing globally. According to the most recent report, 603.7 million adults are obese and approximately 4 million deaths annually should be attributed to obesity and its complications, with the majority of them being due to cardiovascular causes [13]. In the general population, CVD is also the leading cause of mortality, claiming 17.92 million deaths annually [14]. The understanding of the economic and public health benefit that would be yielded by prevention programs has shifted the interest towards interventions for primary prevention of CVD [15,16,17]. This review aims to assess the role of vitamin D in the development of CVD and discuss the impact of obesity as a possible explanation for the discrepancy between retrospective studies and randomized trials.

## 2. Links between Vitamin D Status and Cardiovascular Disease 

### 2.1. Early Evidence from Association Studies and Experimental Data

Over the last decade, it was concluded that patients with coronary artery disease have decreased concentrations of vitamin D [18] and also that patients with decreased concentrations of vitamin D have elevated risk for major adverse cardiovascular events (MACE) [19]. The deleterious effects of vitamin D deficiency at this point were shown to be independent of other vascular comorbidities as hypertension and smoking [20]. Several meta-analyses that have been conducted demonstrate an inverse relationship between vitamin D concentrations and cardiovascular mortality [21], which was independent of the origin of the participants, season of the measurement, and patient’s sex [22]. Moreover, vitamin D deficiency was associated with a decrease of high-density lipoprotein concentration and an increase of low-density lipoprotein (LDL) concentration compared to patients with optimal measurements (134.0 to 131.3, *p* < 0.001) [23]. Experimental data enhanced this concept, as it was demonstrated that vitamin D controls cholesterol concentrations through changes in the activity levels of cytochrome P450 CYP27A1 and induction of cell cycle arrest in the recruited macrophages [24]. Moreover, through direct interaction with nuclear factor kappa beta (NFkB) [25], vitamin D deficiency triggered inflammation both in epicardial fat [25] and in vascular wall, further augmenting the inflammatory response that has been deemed as detrimental for the progression of coronary artery disease and the fragility of the atheroma [26]. At the level of the arterioles, vitamin D concentrations affect the contractility of the muscular layer, and its deficiency increases vascular rigidity [27]. Mice that were fed an atherogenic diet that was deficient in vitamin D demonstrated increased vascular calcification [28]. Regarding the myocardium, Sunbul et al. demonstrated that baseline vitamin D concentrations correlate with echocardiography-measured epicardial fat thickness and global longitudinal strain of the left ventricle, and both parameters correlate well with future history of CVD [29]. At the molecular level, vitamin D was demonstrated to alleviate oxidative stress in the myocardium of animals that were fed a high-fat diet [30] and the adverse effects of advanced glycation end products in the vascular wall of diabetic rats [31]. Thus, a plausible hypothesis has been developed, linking vitamin D deficiency with the pathogenesis of CVD and posing vitamin D as an ideal candidate for the primary prevention treatment of CVDs [32].

### 2.2. Prospective Studies and Randomized Clinical Trials

Unfortunately, the anticipation that was created by the encouraging results of laboratory and association studies has fallen short. Several meta-analyses were conducted but did not show any difference between patients treated with vitamin D and those who were not [33]. Postmenopausal women (*n* = 36,282) from the Women’s Health Initiative participated in a prospective randomized clinical trial to assess the effects of calcium and vitamin D supplementation. When assessing cardiovascular outcomes, no difference was uncovered in MACEs between women that were treated with vitamin D and those that were treated with placebo [34]. A post-hoc analysis, 11 years after termination of the study, also did not confirm benefits from vitamin D supplementation regarding CVD [35]. However, only prospective randomized clinical trials could effectively assess whether the vitamin D hypothesis is right or wrong [33]. A prospective clinical trial in New Zealand that involved 5108 participants and assessed the impact of vitamin D supplementation in the primary prevention of MACE, arrhythmia, and vein thrombosis did not show any difference in the incidence of these disorders, which was similar in both placebo and intervention arms. Only 25% of the participants were vitamin D-deficient, and only 2% had a 25(OH)D concentration <10 ng/ml [36]. The vitamin D and Omega-3 Trial (VITAL) was designed to assess whether vitamin D supplementation could represent an effective mean of primary prevention of both cancer and CVD. Despite the large study population of 25,871 participants and the adequate dosing of 2.000 international units (IU) of vitamin D, the trial did not demonstrate any difference in outcomes between the two treatment groups in terms of both mortality and MACE. That was unchanged even after adjusting for confounding risk factors, including body mass index (BMI); the groups, however, were divided in those with BMI < 27.1 and those with BMI ≥ 27.1 kg/m^2^ [37]. Last but not least, in a trial assessing a population of adult diabetics, vitamin D supplementation did not ameliorate other vascular comorbidities such as blood pressure, with only modest changes in pulse wave velocity [38], and the same outcomes were confirmed from another study in Europe in which patients diagnosed with hypertension were treated with vitamin D but did not show any improvement in their blood pressure [39]. In a large study involving 68,132 post-menopausal women, supplementation of calcium and vitamin D did not result in any change in low-density lipoprotein concentration, which remained unchanged after adjusting for dosing [40]. Even when shifting from the general population to frail adults, the results are still not encouraging, as all-cause mortality in patients with heart failure was not affected by vitamin D supplementation. Moreover, the initial vitamin D measurement did not have any effect on death rates [41]. In patients with metabolic syndrome, vitamin D supplementation could not reverse LDL-cholesterol concentrations, hemoglobin (Hb)A1c, and diastolic blood pressure [42].

## 3. The Inter-Relationship between Obesity, Vitamin D, and Cardiovascular Health

### 3.1. Effects of Obesity on Vitamin D Deficiency and Cardiovascular Disease

Could these risk factors act as confounders and be the reason for the failure of vitamin D in the prevention of CVD despite the reports from observational studies? It is well known that confounders are a paramount limitation in cross-sectional analyses and limit their validity [43]. Results from observational prospective longitudinal studies indicate obesity as a probable confounder. The analysis of the serum from 1484 children in Denmark demonstrated an odds ratio (OR) of 3.41 for vitamin D deficiency in obese individuals and an inverse relationship between vitamin D concentrations and BMI [44]. The relationship was even more profound in infants, as 70.9 to 88.4% of obese children aged 1 to 5 years in Poland were demonstrated to have either suboptimal or deficient vitamin D concentrations [45]. In adults, the association is also significant, yet of lesser magnitude, with obese adults of Mexican descent presenting a 78% increased risk for developing vitamin D deficiency compared to individuals with normal BMI [46]. In the United States, obesity was recognized as an important risk factor for the development of vitamin deficiency, as obese patients had a 58% probability of developing it, compared to 33% for individuals with normal weight [47]. In Europe, a cross-sectional analysis of the adult population in the city of Porto demonstrated that obese individuals had lower vitamin D concentrations independently of the season in which the measurement took place [48]. In a Serbian study, 88% of obese individuals had vitamin D deficiency compared with only 31% of controls [49]. Similar results were reported in Asian populations, as body fat had a negative association with serum vitamin D concentrations [50,51].

On the other hand, obesity also increases the incidence of CVD in adults substantially. Peak BMI has been demonstrated to be detrimental for survival in a prospective study that included 457,785 men and 588,369 women that were followed up for 14 years [52]. In a large retrospective study that was assembled in the United Kingdom and consisted of 3.5 million adults, obesity was associated with a 49% increase of the risk for coronary heart disease in 5.4 years of follow-up [53]. Non-smoking adults were found to have a dose-dependent increase in cardiovascular mortality compared to their peers with normal weight (hazard ratio 2.04, 3.05, and 4.42 for BMI of 30–34.9, 35–39.9, and 40–49.9 kg/m^2^, respectively) [54]. Obese adults were demonstrated to have progression of atherosclerotic lesions even after receiving optimal medical treatment with statins for hyperlipidemia [55]. The presence of obesity along with diabetes mellitus and hypertension resulted in an increase in the incidence of both mortality and heart failure, with expected benefit from prevention of obesity [56]. Moreover, other indicators of excessive adiposity such as the visceral adiposity index have also been identified as independent predictors of 10-year risk of CVD in a prospective clinical study that involved 3042 Greek adults [57]. Apart from the outcomes, obesity also markedly affects other conventional cardiovascular risk factors, including systolic blood pressure [58] as well as oxidized LDL-cholesterol concentrations [59]. Moreover, the prevalence of diabetes mellitus is markedly increased in obese adults compared to individuals with normal BMI, and its incidence strongly correlates with the incidence of obesity in the population [60].

### 3.2. Pathophysiology of Vitamin D Deficiency in Obesity

The pathophysiology link between obesity and serum vitamin D concentrations is complex, yet well established. Obese patients have decreased serum 25(OH)D concentrations compared with healthy controls after oral supplementation of vitamin D [61]. However, the peak levels just after ingestion was the same between the two groups; this formulated the hypothesis that vitamin D is stored in the adipose tissue and, thus, its bioavailability for conversion to its active metabolites is lost [61]. In agreement with this notion, animal studies showed that 25(OH)D was stored for 33% in fat and for 20% in muscle [62]. Thus, muscle might act as another reservoir of vitamin D in obese humans, who have been documented to have an adaptive increase in lean body mass [63,64]. On the other hand, Jung et al. [65] documented an increase of 1,25(OH)2D in mice that were fed a high-fat diet, which could be attributed to increased mRNA levels of renal 1-hydroxylase. In the same experiment, the authors demonstrated that the adipose tissue of these animals also had increased levels of vitamin D receptor, further enhancing the hypothesis of storage in adipose tissue. In another mouse study, [66] obese animals that were fed a high-fat diet had similar vitamin D and 25(OH)D concentrations but decreased plasma concentration of both 25(OH)D free form and vitamin D compared with controls. In this study, renal 1-hydroxylase concentrations were decreased in the obese animals [66]. Perhaps, the reason for the different outcomes might be the composition of the fatty acids, as it was recently demonstrated that renal 1-hydroxylase expression is induced by mono-unsaturated fatty acids but inhibited by saturated fatty acids. Thus, it is not only the amount of consumed fat but also the composition of fatty acids in the adipose tissue that play an important role in the response of hydroxylases and in determining the availability of the active form of vitamin D [67]. The increased expression of renal 1-hydroxylase was abolished after a change from high- to low-fat diet in the animals, consolidating the causal association between adiposity and 25(OH)D concentrations [65].

Regarding endogenously synthesized vitamin D, an increase in BMI results in a decrease of cholecalciferol synthesis for the same exposure to ultraviolet B irradiation, provided that the other controlling factors, such as the surface of the exposed area, remain stable [68]. Despite this observation, obese individuals had lower sunlight exposure and decreased outdoor activity, which further decreased the biosynthesis of vitamin D [69]. Finally, obese patients have increased risk for hepatic steatosis and non-alcoholic fatty liver disease, which downregulate the formation of 25(OH)D in the liver [70]. In a cross-sectional study, patients with non-alcoholic fatty liver disease had high risk of vitamin D deficiency, which was positively correlated with the severity of the disease [71]. 

### 3.3. Effects of Vitamin D Deficiency on Cardiovascular Health in Obese and Non-Obese Populations

The above-presented evidence should not lead to the conclusion that vitamin D deficiency does not have detrimental consequences for vascular health. However, both the prevalence of vitamin D deficiency and its effects are exponentially increased in obese people, leading to the question of choosing the correct population. In obese rats, a vitamin D-deficient diet led to the suppression of beta-oxidation and the accumulation of macrophages due to adipose tissue inflammation [72]. In a rodent model of induced hypertension, treatment with vitamin D ameliorated the outcomes and resulted in a decrease in blood pressure [73]. In another rodent model, deficiency of vitamin D resulted in exacerbation of the hypertension phenotype, fostering the association between vitamin D and blood pressure regulation [74]. Treatment with vitamin D resulted in major differences in the degree of vascular inflammation in a porcine model of hypercholesterolemia that underwent primary coronary intervention [75]. In obese and overweight humans, vitamin D supplementation resulted in the decrease of urinary isoprostane and of the vascular augmentation index, underlining prospective benefits for vascular health and oxidative stress [76]. However, in all these models (Table 1), the animals were not healthy but had another prominent cardiovascular risk factor. In all vitamin D supplementation studies, the target was the general population. The most definitive answer was provided by a meta-analysis of 42,024 adults pooled from 21 cohort studies. The authors performed a bi-directional Mendelian randomization analysis in which they compared the incidence of vitamin D deficiency in haplotypes associated with obesity to the incidence of obesity in haplotypes associated with vitamin D deficiency. The results indicate that, when correcting for environmental confounders, there is a strong negative correlation between BMI and vitamin D status and that obese adults are expected to have vitamin D concentrations <50 nmol/mg, hence explaining the extreme incidence of vitamin D deficiency in obese adults [77]. The paradigm of differential effects of vitamin D deficiency according to the general health of the population is not a novel concept. While vitamin D concentrations have been found to correlate with oxidative stress both in obese children [78] and in elderly diabetic individuals [79], when measured in a healthy population, the association was either very weak or non-existent [80].

## 4. Future Perspectives

**Figure 1:**
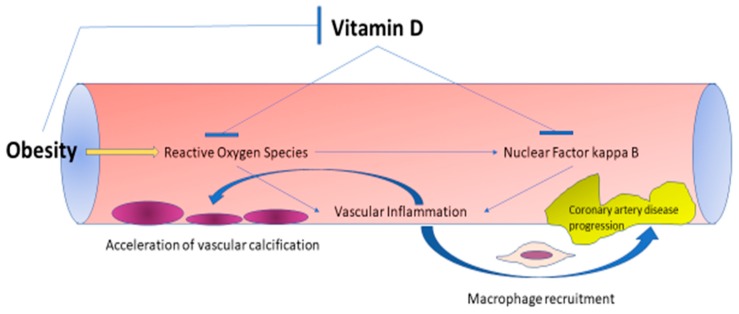
Proposed effects of vitamin D on obesity-related atherosclerotic progression. Vitamin D prevents further macrophage recruitment in atherosclerotic lesions and decreases vascular inflammation through inhibition of nuclear factor kappa B and decoying of reactive oxygen species. Obesity alleviates the beneficial effects of vitamin D on the vasculature by decreasing vitamin D bioavailability and augmenting the production of reactive oxygen species.

Consolidating these findings, we can speculate that CVD occurs in response to an underlying condition which is then further exacerbated by vitamin D deficiency, most notably due to the lack of its countereffect on oxidative stress (Figure 1). There is strong evidence to support that obesity could well constitute this underlying confounder due to its high prevalence and its detrimental effect on both vitamin D status and cardiovascular mortality. Therefore, we could think that the adoption of the general population as the target for prevention of CVD with vitamin D supplementation was the probable cause for the failure of trials in demonstrating benefit of vitamin D supplementation for the prevention of CVD and not necessarily a lack of effects of vitamin D in the cardiovascular system. Only a prospective clinical trial including either patients with a particular risk factor or a population with increased general cardiovascular risk could definitively answer whether prevention of CVD was a mistargeted intervention or a misconception.

## Figures and Tables

**Table 1:** Effects of Vitamin D on vascular health in various studies of animal models and clinical research.

First Author, Year	Study Type	Population	Vitamin D Parameter	Outcome	Reported Interaction with Vitamin D
Studies in animals					
Salum, 2013 [31]	Experimental	Diabetic rats	Vitamin D supplementation	Carboxymethylycin accumulation	Negative, significant
Ellam, 2014 [28]	Experimental	Apolipoprotein E knockout mice	Induced deficiency	Atheroma calcification	Positive, significant
Yin, 2015 [24]	Experimental	Hypercholesterolemic swine	Induced deficiency	Macrophage recruitment	Positive, significant
Chen, 2016 [25]	Experimental	Hypercholesterolemic swine	Induced deficiency	NFkB activity	Positive, significant
Chang, 2017 [72]	Experimental	Obese rats	Induced deficiency	Macrophage recruitment	Positive, significant
Farhangi, 2017 [30]	Experimental	Obese rats	Induced deficiency	Superoxide dismutase/Catalase activity	Negative, significant
Hadjadj, 2019 [27]	Experimental	Hyperandrogenic female rats	Induced deficiency	LAD relaxation capacity	Negative, significant
Studies in humans					
Hsia, 2007 [34]	Experimental, clinical	Postmenopausal women	Vitamin D supplementation	Major adverse cardiovascular events / Stroke	Non-significant
Giovanucci, 2008 [19]	Observational clinical	Healthy adult men	Baseline concentrations	Major adverse cardiovascular events	Inverse, significant
Cauley, 2013 [35]	*Post-hoc* experimental, clinical	Postmenopausal women	Vitamin D supplementation	All-cause mortality	Non-significant
Martins, 2014 [76]	Experimental, clinical	Obese Adults	Vitamin D supplementation	Arterial stiffness	Inverse, significant
Schöttker, 2014 [22]	Meta-analysis	General population	Baseline concentrations	Cardiovascular mortality	Inverse, significant
Welles, 2014 [20]	Observational clinical	Stable cardiovascular disease	Baseline concentrations	Major adverse cardiovascular events	Inverse, significant
Sunbul, 2015 [29]	Observational	Healthy adults	Baseline concentrations	Global longitudinal strain	Statistically significant
Faridi, 2017 [23]	Observational clinical	General population	Baseline concentrations	Total cholesterol	Inverse, significant
Scragg, 2017 [36]	Experimental, clinical	General population	Vitamin D supplementation	Cardiovascular disease incidence	Non-significant
Zhang, 2017 [21]	Meta-analysis	General population	Baseline concentrations	Cardiovascular mortality	Inverse, significant
Manson, 2019 [37]	Experimental, clinical	General population	Vitamin D supplementation	Major adverse cardiovascular events / Stroke	Non-significant

NFkB: nuclear factor kappa beta; LAD: left anterior descending artery.
